# Equine Torovirus (BEV) Induces Caspase-Mediated Apoptosis in Infected Cells

**DOI:** 10.1371/journal.pone.0020972

**Published:** 2011-06-15

**Authors:** Ana M. Maestre, Ana Garzón, Dolores Rodríguez

**Affiliations:** Department of Molecular and Cellular Biology, Centro Nacional de Biotecnología, CSIC, Madrid, Spain; Institut Pasteur, France

## Abstract

Toroviruses are gastroenteritis causing agents that infect different animal species and humans. To date, very little is known about how toroviruses cause disease. Here, we describe for the first time that the prototype member of this genus, the equine torovirus Berne virus (BEV), induces apoptosis in infected cells at late times postinfection. Observation of BEV infected cells by electron microscopy revealed that by 24 hours postinfection some cells exhibited morphological characteristics of apoptotic cells. Based on this finding, we analyzed several apoptotic markers, and observed protein synthesis inhibition, rRNA and DNA degradation, nuclear fragmentation, caspase-mediated cleavage of PARP and eIF4GI, and PKR and eIF2α phosphorylation, all these processes taking place after peak virus production. We also determined that both cell death receptor and mitochondrial pathways are involved in the apoptosis process induced by BEV. BEV-induced apoptosis at late times postinfection, once viral progeny are produced, could facilitate viral dissemination *in vivo* and contribute to viral pathogenesis.

## Introduction

Toroviruses are enteric (and probably respiratory) viruses which infect different animal species and humans and cause diarrhea. They are positive-sense, single stranded RNA enveloped viruses that, together with coronaviruses, belong to the *Coronaviridae* family of the *Nidovirales* order [1], [2]. There are four torovirus species recognized by the ICTV, established according to the animal host that they infect: bovine torovirus (BToV), porcine torovirus (PToV), human torovirus (HToV) and equine torovirus (EToV) (ICTV web site: www.ictvonline.org). The latter virus was the first torovirus identified, isolated in 1972 from the faeces of a horse in Berne (Switzerland), and named Berne virus or BEV [3]. For a long time BEV was the only strain of the genus that could be grown in cell culture, and therefore it is the most thoroughly studied at the molecular level, and is the prototype member of the genus. Nonetheless, the propagation of different BToV strains in the human HRT-18 cell line has been recently described [4], [5].

The torovirus genome consists of a single RNA molecule of about 25–30 kb. The 5′ two thirds contain two large and overlapping open reading frames, ORF1a and ORF1b, that code for the replication machinery. The last third of the genome contains four open reading frames, ORFs 2–5, coding for the spike (S), membrane (M), hemagglutinin-esterase (HE) and nucleocapsid (N) structural proteins [6]. In BEV the HE gene is partially deleted when compared with other torovirus strains, though the corresponding mRNA is produced [7].

Since their identification in 1972, toroviruses have been poorly studied, and many issues regarding torovirus infections remain unexplored. Significantly, little is known about the morphological, physiological and biochemical changes that occur in torovirus infected cells. Investigating the complex virus-host interactions is important to understand the basis of torovirus-induced disease. There are a few studies about torovirus pathogenesis that were performed with BToV, since it is the only torovirus that has been successfully propagated in experimental infections. In those studies, gnotobiotic and colostrum deprived calves, infected orally or intranasally with BToV, developed moderate to severe diarrhea. Infected animals were sacrificed sequentially during the early stages of infection, and intestinal tissue samples were observed by light and electron microscopy. Cytopathological changes associated with BToV infection were observed in enterocytes from the lower half of the villi, extending into the crypts throughout the lower small intestine, large intestine and dome epithelial cells [8], [9]. Enterocytes showed signs of severe vacuolar degeneration, necrosis and exfoliation. It was suggested that the primary site of infection is in the crypt cells, and that infected cells migrate up to the villous before being shed as the viral cytopathic effect (CPE) develops [10].

On the other hand, the effects of infection in the host with the closely related coronaviruses have been more thoroughly investigated, especially since the emergence of the human coronavirus causing the severe and acute respiratory syndrome (SARS-CoV). Significantly, occurrence of cell death by apoptosis has been observed during infection with several coronaviruses: the mouse hepatitis virus (MHV) [11], [12], [13], transmissible gastroenteritis virus (TGEV) [14], [15], [16], infectious bronchitis virus (IBV) [17], [18], canine coronavirus (CCoV) [19], [20], feline infectious peritonitis virus (FIPV) [21], equine coronavirus [22], and the human coronaviruses OC43 [23], 229E [24] and SARS-CoV [25], [26]. The induction of apoptosis represents one of the major components of the host antiviral responses. Nonetheless, viruses establish intricate and complex interactions with the host to regulate apoptosis to ensure a successful replication cycle that allows the production and spread of virus progeny to neighbouring cells [27], [28]. In fact, many viruses have been reported to inhibit apoptosis early in infection to facilitate the viral replication and to induce apoptosis at later times postinfection to inhibit the inflammatory response and favour viral dissemination [28], [29], [30].

Two cellular proteins which are key effectors of the initial antiviral response triggered by interferon (IFN) and which have been related to the induction of apoptosis, are the protein kinase R (PKR) and the RNase L. Both proteins become activated by double-stranded RNA (dsRNA), and activation of each of these two proteins results in protein synthesis inhibition and apoptosis [31], [32], [33], [34], [35], [36], [37]. Upon activation, PKR phosphorylates the alpha subunit of the eukaryotic initiation factor 2 (eIF2α) impairing its activity, and then causing inhibition of host-cell translation initiation [38], [39], [40]. On the other hand, RNase L, which is activated by 2′5′oligoadenylates produced upon dsRNA activation of the 2′5′oligoadenylate synthetase, degrades both viral and host single-stranded RNA (ssRNA), including ribosomal RNA [41], [42]. Therefore PKR and RNase L activation effectively inhibits viral and cellular gene expression, and the ultimate consequence of their activation is the induction of apoptotic pathways leading to the removal of the infected cell.

Caspases are intracellular proteases (cysteine-dependent aspartate-specific proteases) that play an essential role during apoptosis. There are two main apoptosis pathways regulated by caspases: the extrinsic or cell death receptor pathway, and the intrinsic or mitochondrial pathway. The former is initiated mainly by binding of an extracellular ligand to a death receptor, which in turn aggregates and recruits, through adapter proteins, procaspase-8 molecules, leading to their activation into caspase-8. The mitochondrial pathway is activated after an intracellular signal such as DNA damage, leading to the release of cytochrome c from mitochondria, which binds to the adaptor molecule apoptotic protease activating factor-1 (Apaf-1), and the complex binds to procaspase-9, promoting its autocleavage into caspase-9.

Both pathways converge at the activation of executioner caspases, namely caspase-3, -6,-7, and -10. Caspase-3 is the primary executioner caspase that cleaves most of the cellular substrates examined [43], leading to the characteristic morphological features of apoptosis, such as nuclear shrinking and membrane blebbing [44].

Both cell death receptor and mitochondrial apoptosis signaling pathways can also intersect at the caspase-8-mediated cleavage of Bid, a proapoptotic Bcl2 family member, which promotes cytochrome c release from the mitochondria, and has been shown to be implicated in apoptosis induced by viruses [45], [46], [47], [48].

Here, we report the first biochemical study of the effects caused by a torovirus in the infected cells, and show that typical morphological and biochemical features of apoptosis are induced in BEV infected cells. For the elucidation of the underlying mechanisms, we examined several apoptotic markers, and we found that both intrinsic and extrinsic apoptotic pathways are involved, being the second likely activated through Bid cleavage by caspase-8. To our knowledge, nothing was known about the torovirus-induced alteration on cellular functions and signaling pathways, which would have a profound impact on cell viability and virus pathogenesis.

## Materials and Methods

### Cells and viruses

Equine dermal (E. Derm) cells (NBL-6, ATCC CCL-57), and human fetal lung fibroblasts (MRC-5, ATTC CCL-171), kindly provided by R. de Groot (Utrecht University, Utrecht, The Netherlands) and R. E. Randall (University of St. Andrews, Scotland, UK) respectively, were cultured at 37°C, 5% CO_2_ and 98% humidity, in Dulbecco's modified Eagle's medium (DMEM; Invitrogen, corp) supplemented with 15% and 10% fetal calf serum (FCS) respectively, non-essential aminoacids (1%), gentamicin (50 µg/ml), penicillin (100 U/ml), streptomycin (100 µg/ml) and fungizone (0.5 µg/ml).

Equine torovirus BEV (strain P138/72) [3] was obtained from R. de Groot (Utrecht University, Utrecht, The Netherlands). The virus was grown in E. Derm cells and was partially purified by ultracentrifugation over 20% sucrose cushion. The virus was titrated by plaque assay as previously described [49].

### Ultraviolet (UV) inactivation of BEV

A preparation of partially purified virus was placed in an open tube under an UV lamp (PHILIPS 15W/G15 T8) inside a laminar flow hood, and maintained exposed to UV light for 30 min. Viral inactivation was confirmed by titration of the same viral sample before and after UV irradiation.

### Phase-contrast microscopy

E. Derm cells were mock-infected and infected with BEV [5 plaque-forming units (pfu)/cell] in 12-well plates. At 24 and 48 hours postinfection (hpi) cells were examined under an inverted phase-contrast microscope (LEICA DMI6000B) with a 10×/0.30 objective, and images were acquired with a digital camera (CCD Hamamatsu Orca R2).

### Electron microscopy

Monolayers of E. Derm cells, mock-infected or infected with BEV (5 pfu/cell), were fixed in situ 24 hpi with a mixture of 4% paraformaldehyde (PFA) and 2% glutaraldehyde in 0.1 M phosphate buffer pH 7.4 for 1 h at room temperature. Fixed cells were removed from the dishes, washed in phosphate buffer and processed for embedding in the epoxy resin TAAB-812 (TAAB Laboratories-England UK) by methods previously described [50]. Ultrathin sections of embedded samples were stained with saturated uranyl acetate and lead citrate and examined at 80 kV in a Jeol JEM-1010 (Tokyo, Japan) electron microscope. Pictures were acquired with a Bioscan 792 digital camera (Gatan, Inc.).

### Metabolic labeling

E. Derm and MRC-5 cells were seeded in 24-well plates, and infected with BEV (5 pfu/cell). At the indicated times postinfection, cells were subjected to a 30 min pulse, at 37°C with 100 µCi/ml of [^35^S]Met-Cys (GE Healthcare), and DMEM without Met and Cys. Cell extracts were lysed in Laemmli buffer with 5% β-mercaptoethanol, and proteins were denatured by boiling for 5 min, separated by SDS-PAGE in 12% polyacrylamide gels, and visualized after staining of the gel with coomassie blue. Labeled proteins were detected by autoradiography of the dried gels.

### Apoptosis quantitation by analysis of the nuclear DNA content by flow cytometry

The different stages of cell cycle and the percentage of cells with subG_0_-G_1_ DNA content were analyzed by propidium iodide (PI) staining and flow cytometry [51]. E. Derm and MRC-5 cells were seeded in 6-well plates, and infected with BEV (5 pfu/cell). In some experiments, after virus adsorption, cells were treated with the caspase inhibitors z-IETD-fmk (caspase-8), z-LEHD-fmk (caspase-9) and z-VAD-fmk (general inhibitor) (Calbiochem), each at a final concentration of 50 µM. At the indicated times, all the cells from each well were collected; the floating cells were taken with the medium and combined with those still adhered to the plastic recovered by trypsinization. Cells were washed with ice-cold phosphate-buffered saline (PBS), and permeabilized with 70% ethanol in PBS at 4°C overnight. Later, cells were washed three times with PBS, incubated 45 min at 37°C with RNase-A (0.1 mg/ml) and stained with PI (10 µg/ml). The percentage of cells with hypodiploid DNA content was determined by flow cytometry. Data were acquired on 10^4^ cells per sample in a Coulter EPICS XL-MCL cytometer with Argon 488 laser.

### Immunofluorescence

For nuclei staining of apoptotic cells, E. Derm and MRC-5 cells were seeded over coverslips, infected with BEV (5 pfu/cell), and fixed with 4% PFA at the indicated times. Cells were stained with Hoechst 33258 (0.5 µg/ml, Sigma), and the images were obtained in a Leica DMRD epifluorescence microscope, using the suitable filter (360–400 nm).

### rRNA analysis

To analyze rRNA degradation, E. Derm and MRC-5 cells were seeded in 6-well plates, and infected with BEV (5 pfu/cell). At 8, 16, 24 and 48 hpi, medium was removed, cells were washed with PBS and lysed with 350 µl of the RLT buffer from the RNeasy Mini kit (Qiagen), and RNA purified following the manufacturer's instructions. Finally, RNA was eluted with 30 µl of diethyl pyrocarbonate-treated water, and 2 µl of each RNA sample was analyzed in 1% agarose gel and stained using ethidium bromide (EtBr, 0.5 µg/ml), under RNase free conditions.

### Subcellular fractionation

E. Derm and MRC-5 cells grown in 6-well plates were mock-infected or infected with BEV at a multiplicity of infection of 5 pfu/cell and collected at 24 or 38 hpi. Cells treated with staurosporine (500 nM) for 6 h were included in the assay as a control of cytocrome c redistribution in cells undergoing apoptosis induced through the mitochondrial pathway. At the indicated times cells were collected in PBS and subjected to digitonin permeabilization as previously described [52]. Briefly, pelleted cells were resuspended in 100 µl of an isotonic sucrose buffer (250 mM sucrose, 10 mM Hepes, 10 mM KCl, 1.5 mM MgCl_2_, 1 mM EDTA, and 1 mM EGTA, pH 7.1) containing 0.05% digitonin (Sigma), and after 2 min at room temperature disrupted cells were centrifugated at 14.000 rpm 5 min, and the soluble cytosolic fractions were transferred to new tubes containing 25 µl of 5× Laemmli sample buffer, and the digitonin-insoluble fractions (membrane-bound organellar fractions) were washed once with isotonic buffer and directly disrupted in 125 µl of 1× Laemmli sample buffer.

### Western blot analysis

Mock-infected or BEV-infected E. Derm and MRC-5 cells were lysed at the indicated times postinfection directly in Laemmli buffer with β-mercaptoethanol, or were subjected to subcellular fractionation as described above, and proteins were denatured by boiling for 5 min. Protein lysates were fractionated by SDS-PAGE and transferred to nitrocellulose membranes (Protran, Schleicher&Schuell) in a semidry blotting apparatus (BioRad) for 45 min at 200 mA. Membranes were blocked for 1 h in PBS containing 5% nonfat dry milk and then probed with different antibodies: rabbit polyclonal anti-PARP (1∶500, Cell Signaling), rabbit polyclonal anti-eIF4GI, that recognizes the amino (N) and carboxy (C) terminal moieties of the eIF4GI protein (1∶5000, kindly provided by Dr. Carrasco from CBMSO, Madrid, Spain), polyclonal anti-phosphorylated (Ser51) eIF2α (eIF2αP) (1∶1000, Biosource), polyclonal anti-eIF2α (1∶500; Santa Cruz Biotechnology), rabbit polyclonal anti-phosphorylated PKR (Thr451) (1∶1000, Invitrogen), monoclonal anti-murine PKR (B-10, 1∶1000; Santa Cruz Biotechnology), mouse monoclonal anti-β-actin (Ab8226, Abcam), rabbit polyclonal against Bid (1∶500, kindly provided by Dr. Yin from Indiana University, Indianapolis, EEUU), a mouse monoclonal anti-cytochrome c (1∶500; Santa Cruz Biotechnology), two rabbit polyclonal sera against the BEV nucleocapsid protein (anti-BEV-N, 1∶1000) and membrane protein (anti-BEV-M, 1∶1000) respectively [53], and a mouse monoclonal anti-S antibody (1∶250). Proteins were detected using horseradish peroxidase-labeled secondary antibodies (Sigma) and an enhanced chemiluminescence Western blot detection kit (ECL, GE Health Care).

### Statistical analysis

Statistical analyses were performed using Student's two tailed t test. Means ± standard deviation for each sample are shown.

## Results

### Morphological appearance of BEV infected cells

**Figure 1:**
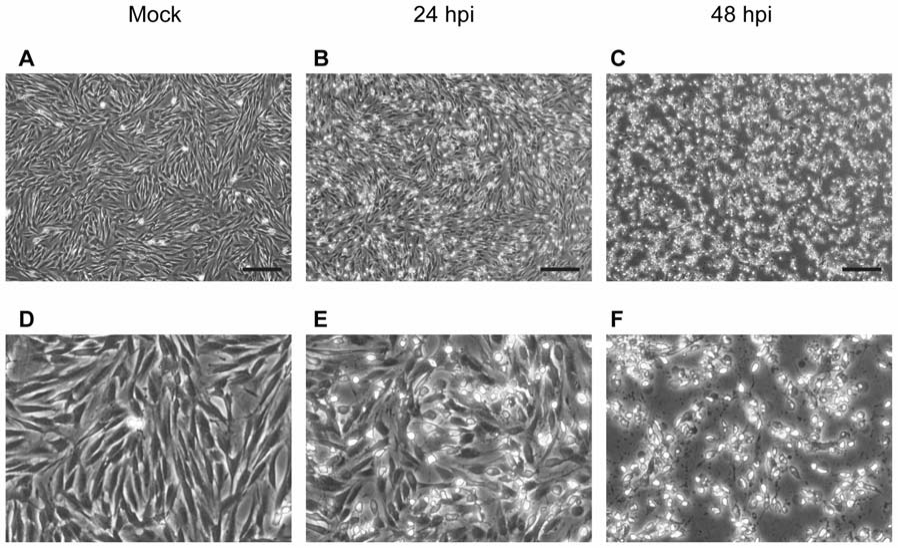
BEV induced CPE in E. Derm cells. E. Derm cells were mock-infected (A and D) or infected with BEV (5 pfu/cell), observed by phase-contrast microscopy and photographed at 24 (B and E) and 48 (C and F) hpi. Panels D, E and F represent enlarged areas (3×) of panels A, B and C respectively. (Scale bars, 200 µm).

**Figure 2:**
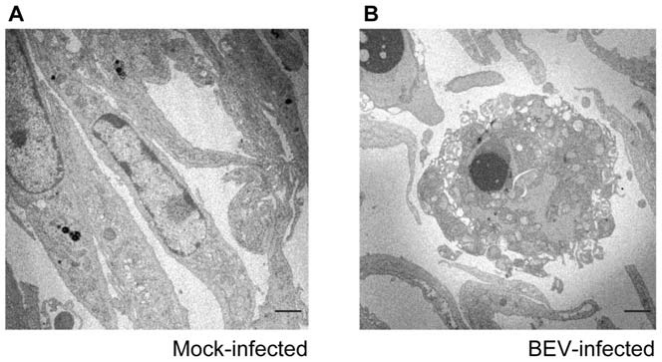
Electron microscopy of BEV infected E. Derm cells. E. Derm cells were mock-infected (A) or infected with BEV (5 pfu/cell) (B), harvested at 24 hpi, fixed and embedded in Epoxi resin. Ultrathin sections of 60–70 nm were stained with lead citrate and uranyl acetate and observed by electron microscopy. Characteristic signs of apoptosis, such as chromatin condensation, membrane blebbing and cell disassembly, observed in the cell of panel B, were evident in some cells infected with BEV at 24 hpi. (Scale bars, 2 µm).

Since little is known about the host cellular responses to torovirus infection, we decided to examine cellular responses to infection with BEV virus, as the prototype member of the torovirus group. Until very recently, BEV was the only torovirus strain that could be grown in cell culture, and E. Derm and embryonic mule skin (EMS) cells the only cell lines described as being susceptible to infection to date. In E. Derm cells infected with BEV at a multiplicity of infection of 5 pfu/cell, the first signs of infection could be detected by about 24 hpi, and CPE was seen in some cells (compare panels A and D with B and E in figure 1). However, extensive CPE could be observed at 48 hpi (Fig. 1, panels C and F). On the other hand, examination by electron microscopy of E. Derm cells infected for 24 h with BEV (5 pfu/cell) showed that some cells presented characteristic signs of apoptosis. Figure 2 shows the comparison of the appearance of an uninfected E. Derm cell (Panel A) with a BEV infected cell showing apoptotic characteristics such as a disorganized nucleus, chromatin condensation, membrane blebbing, and cell disassembly in vesicles (Panel B) [54]. The number of cells showing this morphology readily increases with the time postinfection (not shown), in agreement with the CPE observed by phase-contrast microscopy. These results suggest that BEV causes apoptosis at late times of the infection cycle.

### Protein synthesis inhibition in E. Derm and MRC-5 cells infected with BEV

**Figure 3:**
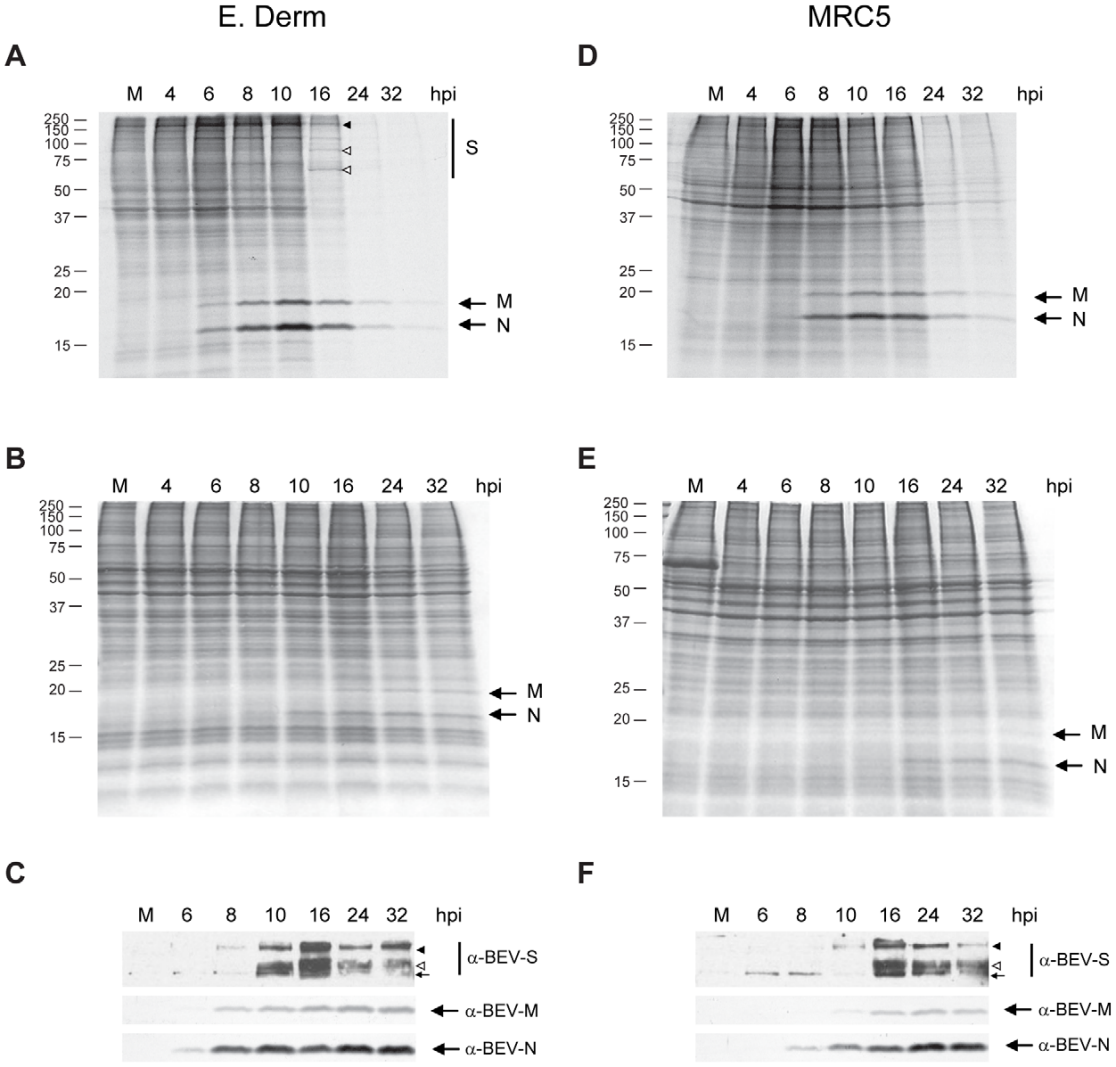
Inhibition of protein synthesis in BEV infected E. Derm and MRC-5 cells. E. Derm and MRC-5 cells were mock-infected (M) or infected with BEV (5 pfu/cell), and metabolically labeled with [^35^S]Met-Cys for 30 min at the time postinfection indicated on the top of the figures. Cellular extracts were analyzed by SDS-PAGE (12% polyacrylamide), and proteins were visualized after staining of the gels with coomassie blue (B and E), and labeled proteins were detected by autoradiography of the dried gel (A and D). The two major proteins that arise throughout the infection with BEV, identified as the structural M and N proteins, are indicated with arrows at the right side of each figure. A high molecular weight protein band (denoted by a solid arrowhead) and two other faint bands (denoted by empty arrowheads) likely representing the S precursor and cleaved products respectively, were also observed in [^35^S]Met-Cys-labeled extracts from E. Derm cells. The positions of the molecular mass markers in kDa are represented on the left. (C and F) Western blot analysis of E. Derm and MRC-5 cells infected with BEV (5 pfu/cell), collected at the indicated hpi and reacted with specific antibodies against the BEV structural S, M and N proteins (indicated at the right of the corresponding panel). The S precursor (denoted by a solid arrowhead), the 100 kDa cleaved product (denoted by an empty arrowhead), and the position of a protein recognized unespecifically by the anti-S monoclonal antibodies (denoted by an arrow) are indicated in the upper panels.

The response of mammalian cells to a variety of stimuli and stress-inducing conditions that cause apoptosis, such as viral infections, involves a rapid inhibition of protein synthesis [55], [56], [57]. To analyze whether protein synthesis was affected after BEV infection, E. Derm cells were infected with BEV (5 pfu/cell) and metabolically labeled with [^35^S]Met-Cys for 30 min at the indicated times postinfection which ranged between 4 and 32 hpi (Fig. 3A). The proteins were analyzed by SDS-PAGE and the gels were stained with coomassie blue and later subjected to autoradiography. A significant general reduction in protein synthesis was observed at 24 and 32 hpi, although a clear decrease was already observed by 16 hpi. The image of the coomassie blue stained gel shows that similar amounts of protein were loaded in all lanes (Fig. 3B). Significantly, two bands corresponding to proteins of about 17- and 20-kDa of molecular mass could be first appreciated in the autoradiogram at 6 hpi, and the intensity of these bands highly increased by 8 and 10 hpi, but diminished thereafter. These two bands should correspond to BEV structural proteins N and M respectively, although their relative mobility in SDS-PAGE are slightly higher than previously described [58], [59], [60]. The identity of the 17- and 20-kDa proteins was confirmed by analysis of the same samples by Western blot with anti-BEV-N and anti-BEV-M antibodies (Fig. 3C). The patterns of appearance of both proteins were similar to those observed by metabolic labeling (Fig. 3A). Interestingly, although the metabolic labeling studies indicated that M and N synthesis decreases at late times of the infection cycle, these proteins were still detected by Western blot analysis suggesting that these proteins remain stable at late time points. In addition, in Fig. 3A a high molecular weight protein can be observed, the intensity of which increased from 6 to 10 hpi and diminished from 16 hpi onwards (labeled with a solid arrowhead), that could correspond to the 200-kDa S protein precursor [61]. Moreover, two additional faint bands in the range of 75- to 100-kDa (labeled with empty arrowheads) could be observed at 16 hpi, that could correspond to the S cleaved products. By Western blot with a monoclonal antibody that recognizes the S protein we observed the precursor at 8 hpi, and both, the precursor and one of the cleaved products from 10 hpi onwards (Fig. 3C).

To further investigate the effect of BEV infection, we searched for other cell types that could be infected by BEV. Thus, several cell lines of different origins were subjected to an infection trial with BEV, and we found that this virus could also grow in the human MRC-5 cells, though the viral replication cycle is slower in this cell type (Garzón et al., unpublished results). Therefore we carried out a similar experiment to that described above, and performed a metabolic labeling with [^35^S]Met-Cys in MRC-5 cells infected with BEV (5 pfu/cell). An overall protein synthesis reduction similar to that observed in E. Derm cells was also observed in MRC-5 cells at 24 and 32 hpi (Fig. 3D). Again, the intensities of whole protein profiles visualized by coomassie blue staining were similar, indicating that equivalent amounts of proteins were loaded in all lines (Fig. 3E). Also, the 17- and 20-kDa viral proteins were detected, although they showed a delay in synthesis with respect to E. Derm cells, being first detected at 8 hpi. Their identity as BEV N and M proteins, respectively, was also confirmed by Western blot (Fig. 3F). Although in the autoradiogram we could not distinguish the high molecular bands corresponding to the S protein precursor and cleaved products among the cellular proteins in these cells (Fig. 3D), by Western Blot with the anti-S monoclonal antibody we could see the S precursor from 10 hpi, and both precursor and one of the cleaved products from 16 hpi onwards (Fig. 3F).

These results show that, in BEV infected cells, synthesis of both cellular and viral proteins was drastically inhibited at late times postinfection.

### eIF2α and PKR are phosphorylated in BEV infected cells

**Figure 4:**
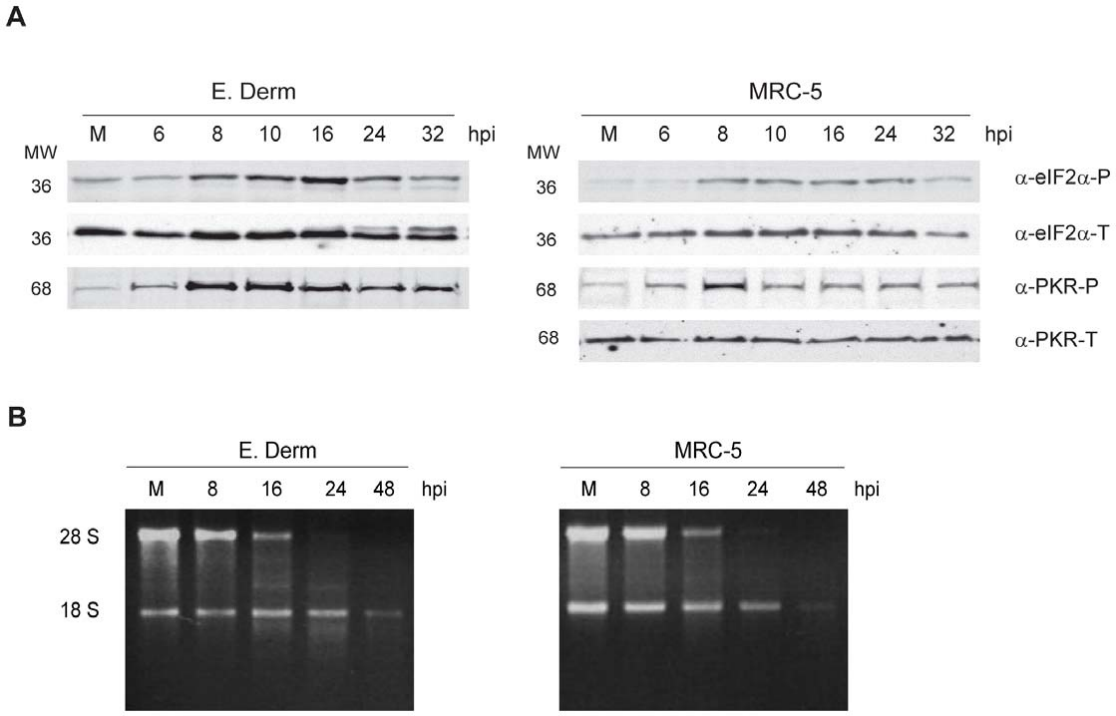
Analysis of factors likely responsible for protein synthesis inhibition. (A) For the analysis of eIF2α and PKR phosphorylation, E. Derm and MRC-5 cells were infected with BEV (5 pfu/cell), collected at different hpi, as indicated on the top of the figure, and subjected to Western blot analysis with antibodies against phosphorylated (α-eIF2α-P) and total (α-eIF2α-T) eIF2α, and phosphorylated (α-PKR-P) and total (α-PKR-T) PKR. The positions of molecular mass markers in kDa are indicated at the left of each panel. (B) For the rRNA degradation analysis, E. Derm and MRC-5 cells were infected with BEV (5 pfu/cell) and collected at the hpi indicated on the top of the figure. RNA from the infected and mock-infected (M) cell cultures was purified and the amount of RNA corresponding to 2×10^5^ cells was fractionated in each lane of a 1% agarose gels stained with EtBr. The positions of the 28S and 18S rRNA molecules are indicated at the left of the figure.

A common mechanism for down-regulating protein synthesis during apoptosis is through the phosphorylation of the translation initiation factor eIF2α [62]. PKR is one of the three Ser/Thr cellular protein kinases known to phosphorylate eIF2α in response to viral infection [33], leading to a block in the translation initiation [31]. Activation of PKR occurs by autophosphorylation upon its binding to dsRNA, which in the case of virus-infected cells is generated as a result of viral replication, or simply as a by-product originated during replication of the viral genome. We have observed that there is accumulation of dsRNA generated as an intermediate product during viral replication in torovirus infected cells (Ávila et al., unpublished results). Hence, we sought to investigate whether eIF2α becomes phosphorylated in BEV infected cells. For that, E. Derm and MRC-5 cells were infected with BEV (5 pfu/cell) and at the indicated times postinfection cells were collected and subjected to Western blot analysis. As shown in figure 4A, a transient eIF2α phosphorylation was observed in both cell lines starting at 8 hpi and decreasing by 24 and 32 hpi. Therefore, next we wanted to determine if PKR could be responsible for eIF2α phosphorylation after BEV infection. Significantly, in both cell lines PKR phosphorylation was first observed at 6 hpi and increased at 8 hpi (Fig. 4A). In E. Derm cells similar phosphorylation dynamics were observed for both eIF2α and PKR. Western blot analysis of these samples with antibodies against total eIF2α and PKR were also performed for sample loading control, however all the antibodies tested against PKR failed to recognize the protein from E. Derm cells.

These results indicate that the protein synthesis blockade observed after BEV infection seems to be related to the phosphorylation of eIF2α, which could be caused by PKR.

### RNA degradation in BEV infected cells

dsRNA is also known to activate the cellular 2′5′-A-dependent RNase L. RNA degradation by RNase L is therefore another cellular mechanism implicated in protein synthesis inhibition at a translational level, and an apoptosis inducer [34], [35], [36], [37]. Hence, we wanted to determine whether rRNA degradation occurs in BEV infected cells, and for this we analyzed 18S and 28S rRNA pattern. rRNA was analyzed in E. Derm and MRC-5 cells infected with BEV (5 pfu/cell) at the indicated times between 8 and 48 hpi. As shown in figure 4B, a similar rRNA degradation pattern was observed in both cell lines, with the 28S RNA being degraded at earlier time points than the 18S; degradation of 18S RNA is only clearly apparent in both cells lines at 48 hpi.. Therefore, these results reinforce the hypothesis that BEV causes apoptosis in infected cells, and suggests that the protein synthesis inhibition is also related to RNA degradation.

### Caspase cascade activation after BEV infection

**Figure 5:**
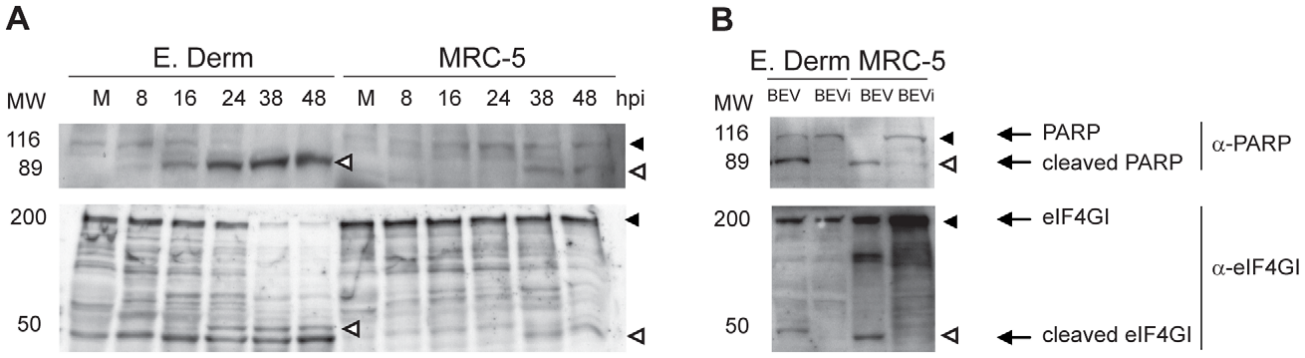
Cleavage of PARP and eIF4GI in BEV infected cells. (A) E. Derm and MRC-5 cells were infected with BEV (5 pfu/cell) and collected at different hpi, as indicated on the top of the figure. As a negative control we used mock-infected cells (M). (B) E. Derm and MRC-5 cells were inoculated with BEV (5 pfu/cell) or UV-inactivated BEV (equivalent amount to 5 pfu/cell) and harvested at 24 (E. Derm) and 38 (MRC5) hpi. (A and B) Cell extracts were fractionated by SDS-PAGE (10% polyacrylamide) and analyzed by Western blot with anti-PARP and anti-eIF4GI antibodies. PARP and eIF4GI full-length proteins are indicated by solid arrowheads and their degradation products are indicated by empty arrowheads. The positions of molecular mass markers in kDa are indicated at the left of each panel.

Caspases, the central executioners of apoptosis, are involved in most of the morphological changes observed in an apoptotic cell. Two well-known substrates of the executioner caspases are the Poly (ADP-ribosylating) Protein 1 (PARP1), a 116-kDa enzyme implicated in the DNA-damage repair, and the eIF4GI initiation factor, the scaffolding protein of the translation initiation complex. Caspase-mediated cleavage of PARP1 produces a 89-kDa C terminal fragment and a 24-kDa N terminal fragment, and renders the protein inactive [63]. The eIF4GI is a 220-kDa protein that can be cleaved in two different places during apoptosis, giving rise to a 76-kDa central fragment and two other fragments of about 50-kDa, corresponding to the N and C termini [64], [65]. To determine whether the caspase-cascade system was activated upon BEV infection, the potential degradation of both factors was studied. E. Derm and MRC-5 cells were infected with BEV (5 pfu/cell), and cell extracts were collected at 8, 16, 24, 38 and 48 hpi. Mock-infected cells were used as a control. Samples were analyzed by SDS-PAGE (10% polyacrylamide gel) and subjected to Western blot analysis with a commercial anti-PARP antibody that recognizes both the 116-kDa intact protein and the 89-kDa fragment, and with a polyclonal antibody that recognizes both the N and C termini of eIF4GI. Appearance of the PARP 89-kDa fragment and a concomitant decrease of the 116-kDa band started between 8 and 16 hpi in E. Derm cells, and at 38 hpi in MRC-5 cells (Fig. 5A, upper panel). eIF4GI presented a similar pattern, with the appearance of the 50-kDa band and the concomitant decrease of the 220-kDa band at 24 hpi onwards in E. Derm cells, and at 38 hpi in MRC-5 cells (Fig. 5A, bottom panel). From these results we concluded that BEV-induced apoptosis involves caspase activation.

We also sought to determine whether virus replication was a prerequisite for virus-induced apoptosis. For this, E. Derm and MRC-5 cells were inoculated with BEV (2.5 pfu/cell) or with an equivalent amount of UV-inactivated BEV. At 24 hpi cells were harvested and analyzed by Western blot with the anti-eIF4GI and anti-PARP antibodies. We did not observe any CPE on the cells treated with UV-inactivated virus (data not shown), and neither of the two caspase substrates was processed in these cells (Fig. 5B), suggesting that BEV apoptosis triggering is dependent upon viral replication.

### DNA degradation in BEV infected cells

**Figure 6:**
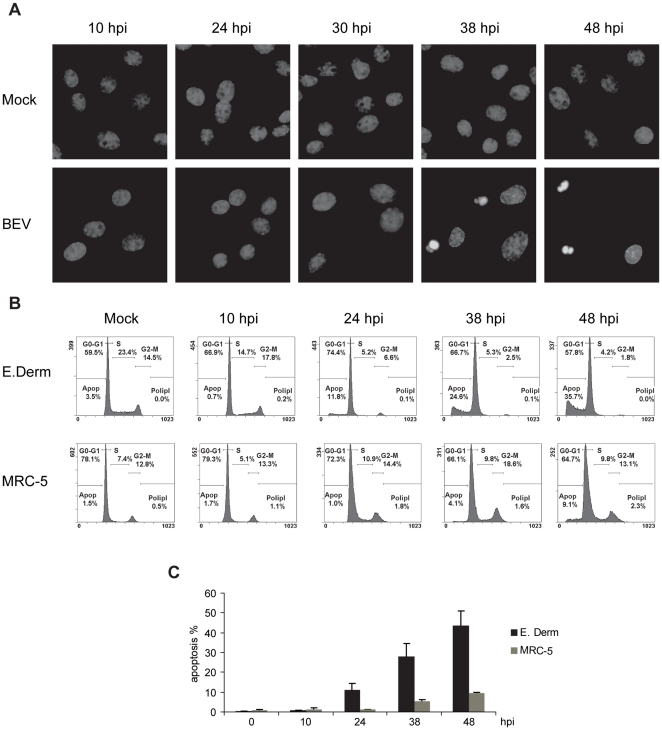
DNA degradation in BEV infected cells. (A) E. Derm cells were infected with BEV (5 pfu/cell) and a nuclei staining kinetic study was performed. As a control, mock-infected cells were used (Mock). Cells were fixed with PFA at the indicated hpi, and nuclei were stained with Hoechst. (B and C) Quantification of DNA degradation in BEV infected cells by cell cycle analysis with flow cytometry. E. Derm and MRC-5 cells infected with BEV (5 pfu/cell) were fixed and PI stained at the indicated hpi. The percentages of apoptotic cells were determined as the subG0-G1 fraction. (B) Cellular cycle graphs obtained in a representative experiment. (C) Graphic representation of the percentages of apoptotic BEV infected cells at different hpi. Data represent the media ± the standard deviation of three experiments.

One of the main characteristics of an apoptotic cell is the nuclear morphological alteration, due to DNA fragmentation, chromatin condensation, and apoptotic body formation [54]. We had previously observed by electron microscopy that, by 24 hpi, the nuclei of about 10% of BEV-infected cells were disorganized, and we wanted to examine the nuclear morphology at later times postinfection. Therefore, E. Derm cells were infected with BEV (5 pfu/cell), or mock-infected, and a kinetic study was performed, fixing cells with PFA at different times between 10 and 48 hpi. Nuclei were stained with Hoechst and observed by fluorescence microscopy (Fig. 6A). While nuclei from control cells were all evenly stained, abnormal brighter and smaller nuclei were instead observed in some BEV infected cells at late times postinfection. At 30 hpi we found a small percentage of apoptotic nuclei (6%), which increases to 18% at 38 hpi. At 48 hpi the percentage of apoptotic nuclei recorded was 12%, but there has to be borne in mind that at this time postinfection many cells were already detached and floating in the culture medium, and therefore, the percentage of apoptotic cells at this time point was underestimated by this approach.

Therefore, in order to quantify more efficiently the kinetics of appearance of apoptotic nuclei, we analyzed the nuclear DNA content by flow cytometry after PI staining. Apopotic nuclei show a reduced fluorescence when compared to non-apoptotic nuclei, and appear as a broad subG_0_-G_1_ peak [51]. Hence, E. Derm and MRC-5 cells infected with BEV (5 pfu/cell) were harvested (monolayers and medium) at 10, 24, 38 and 48 hpi, stained with PI and analyzed by flow cytometry. Fig. 6B shows a representative example of the cell cycle profiles obtained from E. Derm and MRC-5 cells where we could observe a gradual increase in the amount of cells present in the subG_0_-G_1_ peak with increasing time postinfection, although broader peaks were observed in E. Derm cells than in MRC-5 cells. The assay was performed three times, and the results of the three experiments are represented in Fig. 6C. As shown in the figure, 11.17±3.05% of E. Derm cells infected with BEV appear in the subG_0_-G_1_ peak at 24 hpi, and this percentage progressively increased at 38 and 48 hpi (28.13±6.31 and 43.47±7.64% of apoptosis respectively). As observed in previous analysis of protein synthesis and cleavage of caspase substrates, in MRC-5 cells the apoptotic process is delayed, with 5.37±0.97% of apoptotic cells at 38 hpi, increasing to 9.6±0.46 at 48 hpi.

### Both extrinsic and intrinsic pathways are involved in the apoptosis induced by BEV in the infected cells

**Figure 7:**
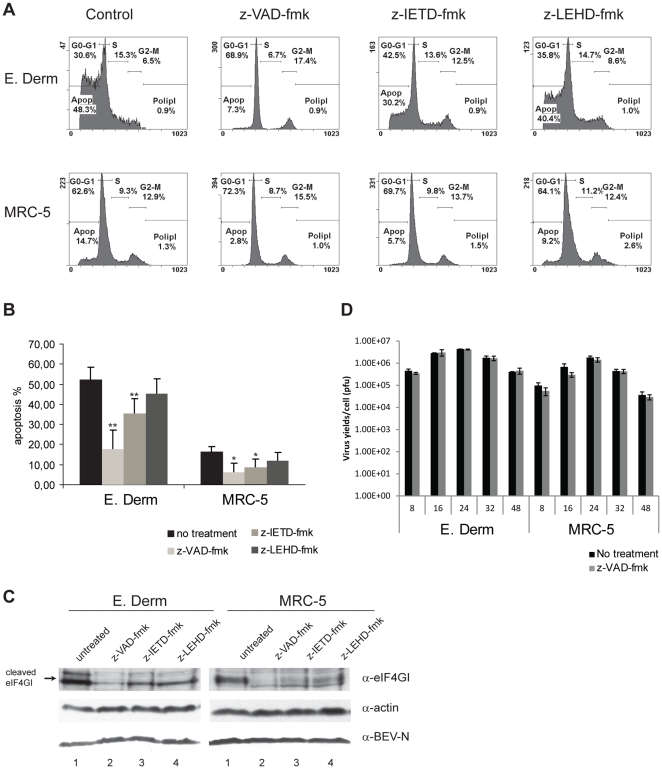
Analysis of the effect of caspase inhibitors z-VAD-fmk, z-IETD-fmk and z-LEHD-fmk on BEV infected cells. E. Derm and MRC-5 cells were infected with BEV (5 pfu/cell) and treated with the general caspase inhibitor (z-VAD-fmk), caspase-8 inhibitor (z-IETD-fmk) or caspase-9 inhibitor (z-LEHD-fmk) (50 µM each). Infected but untreated cells were used as a control (A, B and C). Cells were collected at 48 hpi, and the cellular DNA content was analyzed by flow cytometry after PI staining. (A) Cell cycle graph obtained in a representative experiment. (B) Graphic representation of the percentage of apoptotic cells ± the standard deviation from two experiments. Asterisks indicate statistically significant differences between the means relative to the corresponding control (**p<0.05, *p<0.1; Student's t test). (C) E. Derm cells collected at 24 hpi, and MRC-5 cells collected at 38 hpi were analyzed by Western blot with anti-eIF4GI antibody, and anti-BEV-N and anti-actin antibodies as infection and load control respectively. The position of the cleaved eIF4GI protein is indicated at the left of the panel. (D) E. Derm and MRC-5 cells were infected with BEV at 5 pfu/cell in the absence or presence of z-VAD-fmk, and the supernatants were collected at 8, 16, 24, 32 and 48 hpi and subjected to virus titration. Graphic representation of the media ± the standard deviation of three independent experiments. There were no statistically significant differences between the values in the absence and presence of z-VAD-fmk for any of the two cell lines and at any of the time points tested (p>0.5; Student's t test).

Once we have determined that BEV infection causes apoptosis in both E. Derm and MRC-5 cells, and knowing that the caspase cascade is involved, we wanted to know which upstream caspase(s), and therefore which apoptotic pathway(s) (death receptor and/or mitochondrial pathway) could be involved. For that, E. Derm and MRC-5 cells were infected with BEV (5 pfu/cell), and treated with a general caspase inhibitor (z-VAD-fmk), a caspase-8 inhibitor (z-IETD-fmk), or a caspase-9 inhibitor (z-LEHD-fmk), and the percentage of cells at the subG_0_-G_1_ zone was determined at 48 hpi by flow cytometry after PI staining. The assay was performed twice. It was observed that, in both E. Derm and MRC-5 cells, the general caspase inhibitor clearly protected cells against BEV-induced apoptosis (17.65±9.69 and 6±4.53% of apoptosis respectively in z-VAD-fmk treated cells vs 52.45±5.87 and 16.4±2.40 in non-treated cells), while the other two inhibitors produced a partial reduction in the apoptosis levels, being this decrease higher with the caspase-8 inhibitor (35.40±7.35 and 8.70±4.24% of apoptosis respectively) than with the caspase-9 inhibitor (45.50±7.21 and 12±3.96% of apoptosis respectively) (Fig. 7 A and B). For both E. Derm and MRC-5 cells the difference in the level of apoptosis between non-treated cells and cells treated with the inhibitor of caspase-9 was not statistically significant. Nonetheless, by Western blot analysis we observed a clear effect of this inhibitor on the cleavage of the caspase substrate eIF4GI. For that, untreated and inhibitor-treated E. Derm and MRC-5 cells infected with BEV were collected at 24 and 38 hpi respectively. The cell extracts were incubated with the antibody against eIF4GI, and with anti-BEV-N and anti-actin antibodies as infection and load control respectively (Fig. 7C). In untreated, BEV-infected cells, an intense 50-kDa band corresponding to eIF4GI cleaved product was observed (lanes 1), but the protein was almost totally protected from cleavage by the general caspase inhibitor (lanes 2), and partially protected with caspase-8 (lanes 3) and -9 (lanes 4) inhibitors. Interestingly, cleavage of PARP was not significantly prevented by any of the caspase inhibitors, and it was only slightly inhibited when used at a higher concentration (100 µM) (data not shown).

These results, also in agreement with those obtained by observation of infected cells using light field microscopy (not shown), reveal a trend whereby cell damage induced by apoptosis is higher in the untreated cells, followed in first place by cells treated with caspase-9 inhibitor, secondly by cells treated with caspase-8 inhibitor, and then by cells treated with the general caspase inhibitor. As a whole, these results indicate that although both cell death receptor and mitochondrial pathways are implicated in the BEV induction of apoptosis, the mitochondrial pathway could be secondarily activated by caspase-8-mediated cleavage of Bid.

### Cytochrome c release from the mitochondria and cleavage of Bid

**Figure 8:**
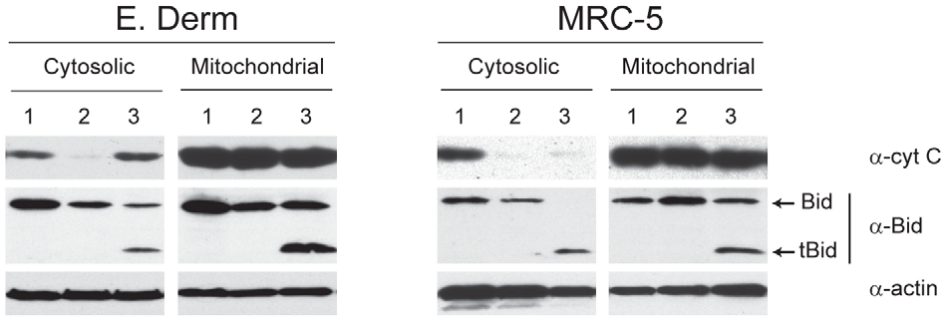
Cytochrome c release from the mitochondria and Bid cleavage upon BEV infection. E. Derm and MRC-5 cells were mock-infected or infected with BEV (5 pfu/cell), harvested at 24 hpi, and fractionated into cytosolic and membrane-bound organellar (mitochondrial) fractions. Cells treated with staurosporine for 6 h, included as a positive control for mitochondrial pathway activation, were also fractionated. Cytosolic and mitochondrial fractions from mock-infected cells (lanes 2), cells treated with staurosporine (lanes 1) or cells infected for 24 h (lanes 3) were analyzed by Western blot with antibodies against cytochrome c and Bid proteins. The positions of the Bid protein and the truncated Bid (tBid) are indicated at the left of the panel B. Reactivity with anti-actin antibody was used as a load control.

To further ascertain the involvement of the mitochondrial pathway in BEV-induced apoptosis we analyzed the cellular distribution of cytochrome c at different times postinfection. For this assay E. Derm and MRC-5 mock-infected cells or cells infected with BEV (2.5 pfu/cell) for 24 h were fractionated into cytosolic and membrane-bound organellar fractions as previously described [52], and analyzed by Western blot. Cells treated with staurosporine were used as positive control of mitochondrial pathway activation. As shown in figure 8 in mock-infected E. Derm cells, cytochrome c was exclusively found in the insoluble membranous fraction (lanes 2), while in BEV-infected cells cytochrome c release to the cytosol could be observed at 24 hpi (lanes 3), and at a similar rate as in staurosporine-treated cells (lanes 1). In MRC-5 cells cytochrome c was clearly present in the mitochondria containing fraction of the differently treated cells, but it could be only barely observed in the cytosolic fractions of cells infected for 24 hpi, while it was clearly observed in the cytosolic fraction of staurosporine-treated cells. This result is in agreement with all the above results that indicate that in MRC-5 cells the apoptotic process occurs at a lower rate (see Fig. 3, 5A, 6B and 6C).

The slight reduction in the percentage of apoptotic cells upon treatment of infected cells with the caspase-9 inhibitor could suggest that the mitochondrial apoptotic pathway may be triggered by caspase-8-mediated cleavage of the pro-apoptotic protein Bid. To study the potential involvement of Bid in this activation we examined Bid proteolytic processing in BEV infected cells. For this, the cellular fractions previously obtained were also analyzed with an anti-Bid antibody. As shown in figure 8, cleavage of Bid into the 15-kDa pro-apoptotic active form (tBid) could be observed in both cell lines infected with BEV (lanes 3). On the other hand, in mock-infected cells (lanes 2) and in cells treated with staurosporine (lanes 1) only the intact 22-kDa form of Bid was detected. These results confirm that the mitochondrial pathway is involved in BEV-induced apoptosis, and points to cleavage of Bid by caspase-8 as the triggering factor.

### Inhibition of BEV-induced apoptosis by the general caspase inhibitor z-VAD-fmk does not affect viral yields

To analyze the potential effect of apoptosis on BEV replication, E. Derm and MRC-5 cells were infected with BEV (5 pfu/cell) in the absence and presence of z-VAD-fmk (50 µM), and supernatants were collected at 8, 16, 24, 32 and 48 hpi. Viral titers were determined by plaque assay. The experiment was performed in triplicate. As shown in figure 7D, there were not significant differences between virus yields obtained at any of the time points analyzed in the absence or presence of the general caspase inhibitor in either cell line. Similar results were obtained when the experiment was performed infecting both cell lines with BEV at a low multiplicity of infection (0.05 pfu/cell) (data not shown). Therefore, this result indicates that caspase-mediated apoptosis is not required for virus replication and/or the release of progeny virions.

## Discussion

Toroviruses have been scarcely studied to date, and hence little is known about both the torovirus effect on the infected cells, and the virus-host interactions established during infection. From studies on pathogenesis caused by BToV in experimentally infected calves, it was described that the virus causes a CPE in the epithelial cells of the intestine, that results in severe vacuolar degeneration, necrosis and exfoliation of the enterocytes, inducing villous atrophy, crypt hyperplasia and in some cases fused villi [66]. On the other hand, initial characterization studies about BEV infection in E. Derm or EMS cells infected *in vitro* showed that cytopathological changes are apparent at about 21 hpi only in about 10% of the cells [67]. We obtained a similar result in E. Derm cells infected at a multiplicity of infection of 5 pfu/cell, where at 24 hpi only some cells show characteristic cythopatic features as cell rounding and detachment. However, extensive CPE was observed at 48 hpi, with many cells floating in the medium. To obtain a more detailed image of the CPE caused by BEV, infected cells were examined by electron microscopy. We observed that, at 24 hpi, some BEV infected cells exhibit morphological changes characteristic of apoptotic cells, such as a condensed nucleus, membrane blebbing, altered mitochondria, and cell disassembly in vesicles. This suggests that BEV infection could induce apoptosis, and this fact could be related to the CPE described in BToV infected animals. The list of viruses that cause apoptosis in infected cells has expanded significantly in the last two decades, and it includes several coronaviruses [14], [18], [19], [21], [22], [24], [68], [69].

To investigate whether BEV actually causes apoptosis we sought to obtain some biochemical evidences. E. Derm cells might not be optimal for investigating host-interactions because it is likely that many of the cellular proteins would not be recognized by available antibodies. Therefore, we used also MRC-5 cells, that we have identified as susceptible for BEV infection. By performing a metabolic labeling in infected E. Derm and MRC-5 cells, we observed that there was protein synthesis inhibition, of both cellular and viral proteins, between 16 and 24 hpi in E. Derm cells, and between 24 and 30 hpi in MRC-5 cells. There are several factors likely to be involved in the induction of this protein synthesis inhibition, common during the apoptotic onset. The generation of dsRNA during genome replication is a usual factor among most RNA viruses, including coronaviruses [70], and in fact, we have observed dsRNA accumulation both in E. Derm and MRC-5 cells infected with BEV (Ávila et al., unpublished data). dsRNA is, in turn, an activator of the innate immune response against viral infections [71]. dsRNA binds and activates two apoptosis triggers that lead to protein synthesis inhibition. On one hand, it activates 2′-5′-oligoadenylate synthetase, leading to activation of latent RNase L and degradation of ssRNAs [34]. Cleavage of rRNA was observed in both cell lines from 16 hpi, suggesting that RNase L could be activated. Cleavage of 28S rRNA was described to occur in MHV infected cells, although in that report the authors showed that the 28S cleavage was an early event in MHV infection that was probably independent of apoptosis, and they argued that another RNase of cellular or viral origin other than RNase L was responsible for this cleavage [72]. In addition, it has been proposed that another as yet unidentified RNase is involved in 28S RNA specific cleavage during apoptosis [73], [74], [75], [76]. Therefore, specific assays will be required to determine whether rRNA cleavage observed in BEV infected cells is mediated by RNase L and/or other ribonuclease. On the other hand, dsRNA also activates endogenous PKR, which leads to eIF2α phosphorylation [38], [39], [40]. Here we show that both PKR and eIF2α become phosphorylated upon BEV infection. However, we cannot discard the possibility that any of the two other virus-activated cellular kinases known to phosphorylate eIF2α, the PKR-like endoplasmic reticulum kinase (PERK) [77] and the general control nonderepressible-2 kinase (GCN2) [78], might be responsible for BEV-induced eIF2α phosphorylation. In this regard, it has been described that, in 293/ACE2 cells, SARS-CoV induces apoptosis via PKR and eIF2α phosphorylation. However, these two events seemed not to be connected, since inhibition of PKR expression prevented apoptosis but not eIF2α phosphorylation. In that case PERK was found to be the responsible kinase [70]. Therefore, the RNA degradation plus eIF2α phosphorylation found in BEV infected cells could contribute to the protein synthesis inhibition upon BEV infection.

The unmistakable sign of the apoptosis induction is the activation of a caspase cascade, which results in the proteolytic cleavage of a series of proteins known to exert important cellular functions [45], [79]. Here we show that cleavage of PARP and eIF4GI substrates readily occur in BEV-infected cells, both E. Derm and MRC-5, in a time-dependent manner. These results also suggest that eIF4GI degradation could be another factor contributing to the observed protein synthesis inhibition. In addition, the nuclei morphological changes characteristic of apoptotic cell death and the loss of nuclear DNA content with time postinfection determined by PI staining and analysis by flow cytometry, are all consistent with the notion that BEV causes caspase-mediated apoptosis.

The study of the apoptotic factors and the nuclear DNA content at several times postinfection indicates that in E. Derm cells the chronology of the processes could be as follows: first PKR and eIF2α phosphorylation (starting at 6 hpi), rRNA degradation, PARP and eIF4GI cleavage by caspases and protein synthesis inhibition (between 16 and 24 hpi), and finally, nuclei degradation (between 24 and 38 hpi). The analysis of most of these parameters in BEV infected MRC-5 cells indicates that in them the apoptotic process occurs at later times postinfection. This observation is in agreement with the lower infection rate in these cells when compared to E. Derm cells, as determined by the lower viral yields (see Fig. 7D, untreated cells) and lower viral protein expression (Fig. 3). Moreover, this result also indicates that caspase-mediated apoptosis requires active BEV replication. In this regard, we could not observe either the characteristic morphological features of apoptosis or the cleavage of PARP and eIF4GI in E. Derm or MRC-5 cells inoculated with UV-inactivated virus. Similar results have been described with SARS-CoV [68], while in the case of MHV it has been reported that viral replication was not required, and it was suggested that virus fusion triggered after virus binding to the cell receptor was sufficient to initiate the apoptosis signal through Fas activation [12].

The analysis of both, DNA content and eIF4GI cleavage, as well as the *de visu* examination of cells infected in the presence of caspase inhibitors, showed that both the intrinsic and extrinsic apoptotic pathways are involved. Moreover, the observed cytochrome c release to the cytoplasm shows that the mitochondrial pathway is involved. Nonetheless, the cell death receptor pathway seems to be predominant in both cell lines since, as determined by the analysis of the nuclear DNA content by flow cytometry, caspase-8 inhibitor (extrinsic pathway) causes about 32% and 47% reduction in apoptosis in E. Derm and MRC-5 cells respectively, compared to reductions of 13% and 27% respectively observed after treatment with caspase-9 inhibitor (mitochondrial pathway). Our results suggest that the mitochondrial pathway can be activated indirectly through cleavage of the pro-apoptotic Bcl2 family member Bid by caspase-8, as it has been shown to occur in CCoV [20] and MHV infected cells [80]. A question arises as to how the extrinsic pathway is activated by BEV infection. In this regard, Fas-mediated signaling has been implicated in apoptosis in response to infection by different viruses (reviewed in [81]). The activation of the Fas pathway may occur extracellularly through the binding of Fas by Fas ligand (FasL) or intracellularly through other signaling pathways. Several studies have indicated that PKR is involved in activating Fas-dependent apoptosis independent of FasL in response to various stress-inducing stimuli, including virus infection [31], [82], [83], [84], [85], [86]. In influenza virus infections, early activation of PKR seems to be important for upregulating Fas gene expression and induction of apoptosis [86], [87]. In this regard, we have observed an early induction of Fas gene expression at the level of transcription upon BEV infection (unpublished results). Thus, the possibility that, as in the case of influenza virus and other viruses, Fas signaling pathway might be responsible for apoptosis induction in BEV infected cells merits further investigation.

Interestingly, PARP cleavage does not seem to be inhibited significantly by any of the caspase inhibitors used. Cleavage of PARP is considered a biochemical hallmark of apoptosis, however it can be regarded as an “special” caspase substrate, since it can be cleaved by both caspases-3 and -7 [79]. Therefore, as opposed to most substrates known to be cleaved during apoptosis by caspase-3, such as gelsolin, α-fodrin, lamin, and probably eIF4GI, PARP can be cleaved in the absence of caspase-3 [43], [88] or in the presence of caspase-3 specific inhibitor [44]. Moreover, it has been suggested that caspase-7 is more efficient than caspase-3 for PARP cleavage [89]. Therefore, it is possible that due to this redundancy, PARP cleavage can still proceed in the presence of caspase inhibitors. Although it has been reported that cleavage of PARP is inhibited in Jurkat cells treated with z-VAD-fmk (100 µM) [90], indicating that this inhibitor also blocks caspase-7, cell-type specific differences, such as the abundance of caspases, could explain this discrepancy. Specific experiments would be required to ascertain which caspase is cleaving PARP in cells infected with BEV in the presence of the general caspase inhibitor z-VAD-fmk.

Our results indicate that apoptosis does not affect virus replication and/or release *in vitro*. We have observed that inhibition of the apoptotic process by treating BEV infected cells, either with low or high multiplicity of infection, with a general caspase inhibitor, does not influence the amount of virus released to the medium. Significantly, in BEV infected cells, maximum viral progeny is already obtained by 24 hpi [67]. Therefore, the virus seems to complete its replication cycle before the onset of apoptosis, a common strategy used by several RNA viruses, and specifically by some coronaviruses, to overcome the effect of premature cell death [14], [18], [20].

In summary, our study shows that BEV infection leads to caspase-dependent apoptotic cell death in E. Derm and MRC-5 cells. The apoptotic process induced by BEV is triggered at late times postinfection, once the viral progeny is produced. However, in torovirus infected animals, apoptosis could favour the viral infection, likely facilitating the viral dissemination and pathogenesis, as suggested with a great number of viruses, mainly RNA viruses that induce apoptosis in the final stages of the replication cycle [27], [28], [91].

The apoptotic death of infected cells causes tissue destruction. In the case of torovirus, the associated gastroenteritis could be related to the apoptosis of the mucosa epithelial cells. In this regard, it has been previously hypothesized for some coronaviruses that apoptosis could be the pathological basis for the lesions of the diseased tissue alteration [19], [92]. Further work will be required to delineate the pathway(s) involved in cell death and to identify the viral and cellular factors involved in modulating torovirus-induced apoptosis. This work can serve as a starting point in the understanding of the virus-host interaction during torovirus infection, a field of research that remains almost unexplored as yet.
